# The impact of volunteering with a student-run free clinic on medical student specialty selection

**DOI:** 10.1186/s12909-022-03788-8

**Published:** 2022-10-11

**Authors:** Kyle B. Thomson, Pranav Mirpuri, Melissa Chen

**Affiliations:** Rosalind Franklin University of Medicine and Science, North Chicago, USA

**Keywords:** Primary care, Family practice, Specialty competitiveness, Student-run free clinic

## Abstract

**Background:**

The shortage of primary care physicians in the United States is expected to grow to 17,800–48,000 physicians by 2034. Student Run Free Clinics are an increasingly popular component of medical schools and may provide an avenue for increasing medical student interest in primary care specialties. However, there is limited research on the impact of Student Run Free Clinics on medical student specialty choice. This investigation sought to determine whether volunteering with the Interprofessional Community Clinic (ICC), the Student Run Free Clinic associated with Chicago Medical School, was associated with an increased likelihood of matching into primary care specialties. Secondarily, the authors investigated associations between volunteering and matching into family practice. Finally, the authors explored associations between volunteering and the competitiveness of specialty choice.

**Methods:**

This retrospective review utilized data on medical school graduates from 2015 – 2021 including their matched specialties, the number of ICC shifts they volunteered for, and whether they held an ICC leadership position (executive officers). Primary care specialties were defined as internal medicine, family practice, pediatrics, or combined internal medicine/pediatrics. Residency fill rate was used as a proxy for competitiveness.

**Results:**

This analysis included 506 medical students (254 ICC volunteers and 252 non-volunteers). Among ICC volunteers, 47.2% matched into a primary care specialty compared to 36.5% of non-volunteers (RR 1.29, 95% CI 1.05–1.59). Each additional shift worked at the ICC was correlated with increased odds of matching into family practice by a factor of 1.042 (95% CI 1.005–1.079).

**Conclusions:**

Medical students who volunteered with the ICC were more likely to match into primary care residencies. Students who volunteered more frequently were more likely to match into family practice. Further investigation is warranted to determine whether these associations are causative and could thus be leveraged to encourage medical students to pursue primary care careers.

## Background

The United Statescurrently faces an acute scarcity of primary care physicians. According to a recent report conducted by the Association of American Medical Colleges (AAMC), this shortage is projected to continue to grow from 13,700 physicians in 2018 to between 17,800 and 48,000 physicians by 2034 [1]. In light of this, it is essential to consider strategies to increase medical student interest in primary care specialties. One such strategy may be through Student Run Free Clinics (SRFCs) at medical schools.

SRFCs are institutions managed and staffed by medical students that aim to provide free medical care to underserved patient populations under the supervision of attending physicians. Students benefit from the increased opportunity for service learning, interprofessionalism, and early exposure to direct patient care while also serving their community [2]. SRFCs are an increasingly popular component of medical schools, with the Society of Student Run Free Clinics currently reporting 152 member institutions [3–5]. However, despite the prevalence of SRFCs, there is limited literature on the impact they have on medical student education. If there is a causative relationship between medical student volunteering with SRFCs (which predominantly provide primary care services) and likelihood of pursuing primary care specialties, then SRFCs may be an invaluable tool for encouraging more medical students to pursue primary care. However, the existence of such an association remains unclear. This investigation sought to determine whether medical student volunteering with the SRFC at our institution was associated with increased likelihood of pursuing primary care specialties.

The Interprofessional Community Clinic (ICC) is the SRFC affiliated with Chicago Medical School (CMS) at Rosalind Franklin University of Medicine and Science. The ICC was founded in 2013 to provide high-quality and equitable healthcare to uninsured patients in the community and encourage future healthcare professionals to work in underserved communities [6]. The ICC operates one day per week for four hours and provides the following outpatient services: primary care, behavioral health counseling, podiatry, and physical therapy. The primary care service is focused on longitudinal patient care and although appointments are required, the occasional walk-in patient is seen. Most patients are followed over several years while others are seen only once. Volunteer opportunities are open to all students at Rosalind Franklin University across multiple disciplines including medicine, pharmacy, podiatry, physical therapy, physician assistant, and behavioral health. Student volunteers attend an orientation and receive extensive training prior to their first shift and are permitted to volunteer throughout their medical school education. On clinic day, student volunteers are grouped into Interprofessional Teams (IP teams) of 5 and are directly responsible for interviewing, examining, and presenting the patient to the attending faculty. IP teams are often led by 3^rd^ and 4^th^ year medical students, but due to time constraints for 3^rd^ and 4^th^ year students, the bulk of student volunteering at the ICC is undertaken by 1^st^ and 2^nd^ year medical students. The ICC is led by student executive officers who oversee week-to-week operations. Executive officers are typically first-year students with leadership experience who are interviewed and selected by previous executive officers. Although the clinic is student led, all clinical care is supervised by licensed faculty.

The primary aim of this study was to investigate whether volunteering with the ICC is associated with an increased likelihood of matching into any primary care specialty. The second aim was to determine if volunteering at the ICC is associated with an increased likelihood of matching into family practice specifically. The third objective was to explore any relationship between volunteering with the ICC and competitiveness of specialty choice. It was hypothesized that some students may pursue volunteering opportunities at the ICC to bolster their residency applications for more competitive specialties. Data from the National Residency Matching Program (NRMP) has shown that residency applicants who match to their preferred specialty have more volunteering experience on average than those who do not [7]. This investigation measured specialty competitiveness using fill rates, in accordance with previous literature on medical specialty competitiveness [8–11].

## Methods

### Study design

This was a retrospective review analyzing ICC shift involvement and specialty choice. Although the clinic opened in 2013, complete archived schedules of all shifts worked at the ICC were only available for Fall 2015 – Spring 2021. These archived schedules were compiled to create a dataset with all students who volunteered during that time. Similarly, publicly available match lists were obtained to create a dataset of all CMS graduates during that same period. These datasets were joined to create a final database including all CMS graduates from 2015 – 2021, the specialty they matched into, the number of shifts worked at the ICC, and whether they were an executive officer at the ICC.

### Sample size and eligibility criteria

The sample size of our study was 506 students. Inclusion criteria were as follows: students had to (1) have had the opportunity to volunteer in the clinic as first-year medical students (graduating classes 2019 – 2021) and (2) have chosen a medical specialty other than “Transitional” or “Preliminary”. However, if the specialty match of students who chose “Transitional” or “Preliminary” could be identified, they were included in the analysis.

**Fig. 1:**
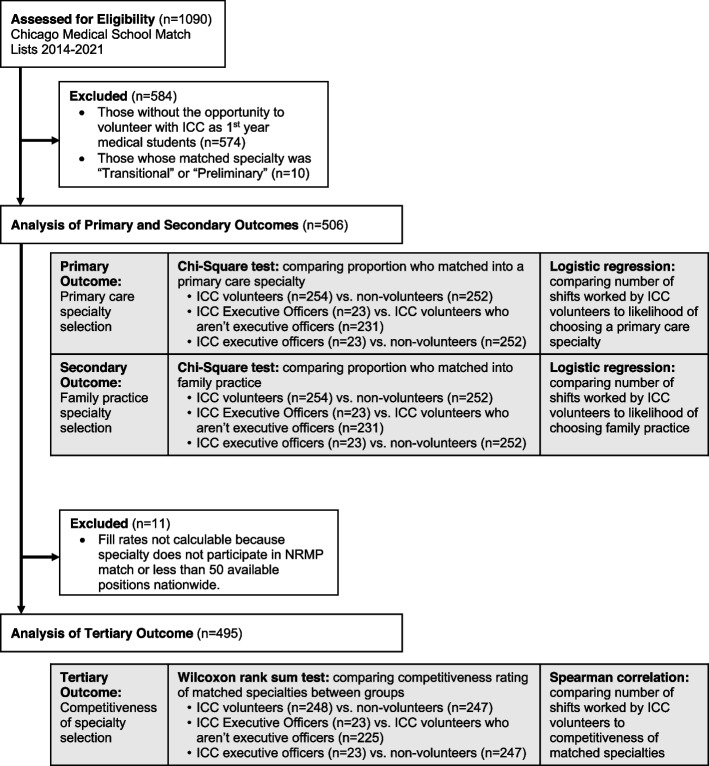
Participant selection flow diagram (white) and outline of statistical procedures (grey)

To analyze competitiveness of specialty selection (tertiary outcome), an additional inclusion criterion (3) was the availability of fill rate data from the NRMP, which was used as a proxy for specialty competitiveness. Fill rates could not be obtained for specialties that did not participate in the NRMP match or for specialties that have less than 50 available positions [13]. Consequently, students who matched into ophthalmology, urology, combined internal medicine/psychiatry, combined internal medicine/emergency medicine, or combined pediatrics/psychiatry/child psychiatry were excluded from the competitiveness analysis. Thus, a further 11 students (2.2%) were excluded in the tertiary outcome analysis for a sample size of 495 students (Fig. 1).

### Outcomes

The primary outcome was the proportion of students who matched into a primary care specialty. Torpy et al. and the American Academy of Family Physicians define primary care as including internal medicine, family practice, and pediatrics. [12, 13]. This is in accordance with how primary care is discussed in NRMP data reports [7]. Therefore, primary care was defined as including the following specialties: internal medicine, family practice, pediatrics, and combined internal medicine/pediatrics. Considering that many medical students who match into internal medicine go on to sub-specialize or provide inpatient care, the outpatient care provided at the ICC is most similar to family practice. As such, the secondary outcome was the proportion of students who matched into family practice specifically.

Finally, the tertiary outcome was the competitiveness of matched specialties. Similar to previous studies, specialty competitiveness was measured using fill rate [8–11]. Fill rate was defined as the percentage of available residency positions filled by US graduates who ranked that specialty first in their rank-order list [7, 9]. Data from the 2020 NRMP Charting Outcomes in the Match report was used to calculate fill rate as a competitiveness rating (Table 1) [7].

**Table 1:** Match results of medical school graduates (2019–2021) by specialty

	**Competitiveness Rating** (Fill Percentage)	**ICC Volunteers**			**Non-ICC Volunteers**	**Total**
		**Executive Officers**	**Non-Executive Officers**	**Total ICC Volunteers**		
		n (%)	n (%)	n (%)	n (%)	n (%)
Plastic Surgery	91.7%	0 (0)	0 (0)	0 (0)	1 (0.4)	1 (0.2)
Otolaryngology	88.6%	0 (0)	2 (0.9)	2 (0.8)	0 (0)	2 (0.4)
Neurological Surgery	87.5%	0 (0)	0 (0)	0 (0)	1 (0.4)	1 (0.2)
Orthopedic Surgery	80.7%	1 (4.3)	4 (1.7)	5 (2)	9 (3.6)	14 (2.8)
Internal Medicine/ Pediatrics^*^	78.7%	0 (0)	3 (1.3)	3 (1.2)	1 (0.4)	4 (0.8)
Obstetrics-Gynecology	75.1%	1 (4.3)	11 (4.8)	12 (4.7)	10 (4)	22 (4.3)
Interventional Radiology	75.0%	0 (0)	5 (2.2)	5 (2)	4 (1.6)	9 (1.8)
Dermatology	72.1%	0 (0)	2 (0.9)	2 (0.8)	5 (2)	7 (1.4)
General Surgery	64.6%	2 (8.7)	9 (3.9)	11 (4.3)	16 (6.3)	27 (5.3)
Emergency Medicine	63.7%	2 (8.7)	27 (11.7)	29 (11.4)	25 (9.9)	54 (10.7)
Anesthesiology	63.2%	1 (4.3)	15 (6.5)	16 (6.3)	26 (10.3)	42 (8.3)
Radiation Oncology	63.0%	0 (0)	0 (0)	0 (0)	2 (0.8)	2 (0.4)
Psychiatry	60.1%	1 (4.3)	11 (4.8)	12 (4.7)	23 (9.1)	35 (6.9)
Diagnostic Radiology	59.3%	0 (0)	11 (4.8)	11 (4.3)	15 (6)	26 (5.1)
Pediatrics^*^	58.4%	0 (0)	26 (11.3)	26 (10.2)	23 (9.1)	49 (9.7)
Child Neurology	57.0%	1 (4.3)	2 (0.9)	3 (1.2)	1 (0.4)	4 (0.8)
Physical Medicine and Rehabilitation	50.2%	1 (4.3)	7 (3)	8 (3.1)	4 (1.6)	12 (2.4)
Neurology	48.4%	1 (4.3)	10 (4.3)	11 (4.3)	12 (4.8)	23 (4.5)
Internal Medicine^*^	39.9%	6 (26.1)	54 (23.4)	60 (23.6)	39 (15.5)	99 (19.6)
Pathology	32.7%	0 (0)	1 (0.4)	1 (0.4)	1 (0.4)	2 (0.4)
Family Medicine^*^	31.3%	6 (26.1)	25 (10.8)	31 (12.2)	29 (11.5)	60 (11.9)
Internal Medicine/ Emergency Medicine	NC	0 (0)	1 (0.4)	1 (0.4)	0 (0)	1 (0.2)
Internal Medicine/ Psychiatry	NC	0 (0)	1 (0.4)	1 (0.4)	0 (0)	1 (0.2)
Ophthalmology	NC	0 (0)	2 (0.9)	2 (0.8)	2 (0.8)	4 (0.8)
Pediatrics/Psychiatry/Child Psychiatry	NC	0 (0)	1 (0.4)	1 (0.4)	0 (0)	1 (0.2)
Urology	NC	0 (0)	1 (0.4)	1 (0.4)	3 (1.2)	4 (0.8)
**Total**	-	23	231	254	252	506

^*^Specialty was considered a primary care specialty

NC: Specialty competitiveness was not calculable because the specialty was not part of the NRMP match, or the specialty had less than 50 available spots

### Independent variables

To investigate the influence of volunteering at the ICC on the outcomes of interest, students who volunteered were compared with students who did not volunteer. In addition, status as an executive officer and the number of shifts worked were used as distinct measures of degree of involvement with the ICC. Students who were executive officers were compared to non-volunteers as well as volunteers who were not executive officers. Associations between the number of shifts worked and the outcomes of interest were assessed (Fig. 1).

### Statistical analysis

Chi-square tests were used to compare the proportions of students who matched into primary care specialties or specifically into family practice. Any chi-squared test that resulted in a significant difference between groups was followed by calculation of a relative risk to determine the magnitude and direction of the difference. To test for associations between the number of shifts volunteered at the ICC and the likelihood of matching into a primary care specialty or family practice specifically, univariate logistic regression models were constructed. Wilcoxon rank sum tests were used to compare the competitiveness rating of matched specialties between groups. Finally, a Spearman correlation was used to test for an association between the number of shifts volunteered at the ICC and competitiveness rating of matched specialties. Figure 1 outlines the statistical tests used for our primary, secondary, and tertiary outcomes. Nonparametric tests were chosen because our data were not distributed normally. All data were analyzed using R statistical software version 4.1.0 (The R Foundation, Vienna, Austria).

## Results

Following application of the exclusion criteria, 506 students were analyzed. Of these, 254 (50.2%) students volunteered with the ICC and 252 (49.8%) did not. A total of 23 (4.5%) students served as executive officers. The 254 student volunteers completed a total of 2,228 shifts. Most of these shifts were completed by first- and second-year medical students (41% and 40%, respectively). The most common specialty choices among the study population were internal medicine (*n* = 99, 19.6%), family practice (*n* = 60, 11.9%), and emergency medicine (*n* = 54, 10.7%) (Table 1).

### ICC volunteering and primary care specialty selection

The proportion of ICC volunteers who matched into a primary care specialty was 47.2% (120 students), which differed significantly from the 36.5% of non-volunteers who matched into a primary care specialty (92 students, *p* = 0.014). ICC volunteers were 29% more likely to match into a primary care specialty than non-volunteers (RR 1.29, 95% CI 1.05–1.59) (Table 2). The proportion of ICC executive officers who matched into a primary care specialty was 52.2% (12 students) and did not differ significantly compared to 46.8% of volunteers who were not executive officers (108 students, *p* = 0.620) or compared to 36.5% of non-volunteers (92 students, *p* = 0.138) (Table 2).

**Table 2:** Between group comparisons of the proportion who matched into primary care, proportion who matched into family practice, and the competitiveness of specialties chosen

**Outcome**	**Statistical Test Used**	**ICC Volunteers**			**Non-ICC Volunteers** *n* = 252	***P*-Value**
		**Executive Officers** *n* = 23	**Non-Executive Officers** *n* = 231	**Total ICC Volunteers** *n* = 254		
Matched into a primary care specialty, *n (%)*	Chi-Square	-	-	120 (47.2)	92 (36.5)	.014
		12 (52.2)	-	-	92 (36.5)	.138
		12 (52.2)	108 (46.8)	-	-	.620
Matched into family practice, *n (%)*	Chi-Square	-	-	31 (12.2)	29 (11.5)	.809
		6 (26.1)	-	-	29 (11.5)	.045
		6 (26.1)	25 (10.8)	-	-	.033
Competitiveness rating of matched specialties, *median (IQR)*	Wilcoxon Rank Sum	-	-	58.3 (23.7)	59.3 (23.7)	.061
		39.9 (27.8)	-	-	59.3 (23.7)	.067
		39.9 (27.8)	58.4 (23.7)	-	-	.164

Note: Only two groups were compared at a time to allow for direct comparisons and because executive officers are not mutually exclusive from ICC Volunteers

In a univariate logistic regression model, the number of shifts worked by ICC volunteers was not significantly correlated with matching into a primary care specialty (*p* = 0.783). For each additional shift worked at the ICC, the odds of matching into a primary care specialty were expected to increase by a factor of 1.004 (95% CI 0.976–1.033).

### ICC volunteering and family practice specialty selection

The proportion of ICC volunteers who matched into family practice was 12.2% (31 students) and did not differ significantly from the 11.5% of non-volunteers who matched into family practice (29 students, *p* = 0.809) (Table 2). The proportion of ICC executive officers who matched into family practice was 26.1% (6 students) and differed significantly compared to the 10.8% of volunteers who were not executive officers (25 students, *p* = 0.033) as well as the 11.5% of non-volunteers (29 students, *p* = 0.045). Executive officers at the ICC were 141% more likely to match into family practice than ICC volunteers who were not executive officers (RR 2.41, 95% CI 1.10, 5.26). Executive officers were 127% more likely to match into family practice than non-volunteers (RR 2.27, 95% CI 1.05–4.89) (Table 2).

**Fig. 2:**
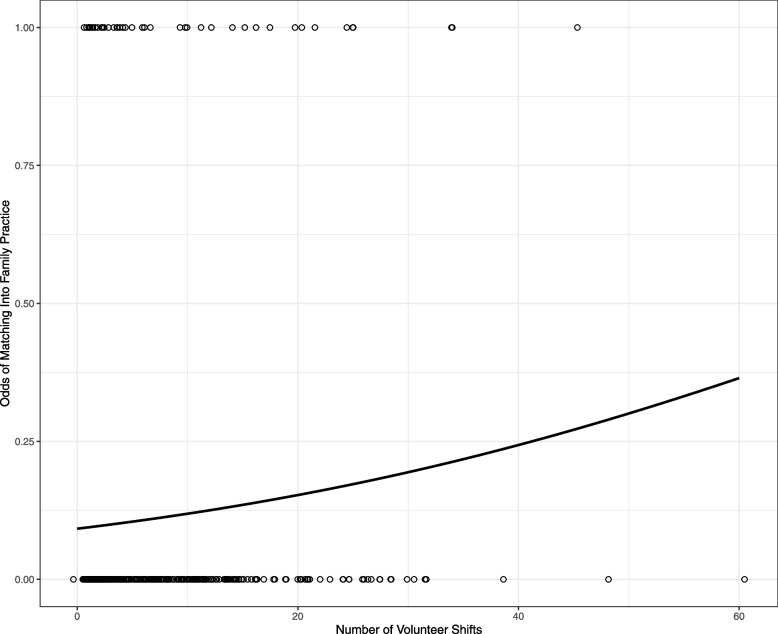
Logistic regression comparing the number of shifts volunteered by medical students to the odds of matching into family practice

In a univariate logistic regression model, the number of shifts worked by ICC volunteers was significantly correlated with the likelihood of matching into family practice (*p* = 0.022). For each additional shift worked at the ICC, the odds of matching into family practice were expected to increase by a factor of 1.042 (95% CI 1.005–1.079) (Fig. 2).

In a post-hoc analysis, executive officers were noted to work more shifts on average (18.7) compared to volunteers who were not executive officers (7.8). To adjust for potential confounding between executive officer status and number of shifts worked, two additional tests were run: (1) the proportion of executive officers matching into family practice compared to non-executive officer volunteers stratified by the number of shifts worked, and (2) multiple logistic regression was completed including executive officer status as well as number of shifts worked.

Among volunteers who worked more than 18.7 shifts, the proportion of executive officers who matched into family practice (33.3%) was no different from non-executive officer volunteers (20.0%, *p* = 0.399). Of those who worked less than 18.7 shifts, the proportion of executive officers who matched into family practice (18.2%) was no different from non-executive officer volunteers (9.9%, *p* = 0.383). In multiple logistic regression, the number of shifts worked remained significantly correlated to the likelihood of matching into family practice (*p* = 0.042), with the adjusted odds of matching into family practice expected to increase by a factor of 1.039 for each shift worked. Executive officer status was not correlated with odds of matching into family practice in the multiple logistic regression (*p* = 0.247).

### ICC volunteering and competitiveness of specialty selection

The median competitiveness rating of specialties that ICC volunteers matched to was 58.3 (IQR 23.7), whereas the median competitiveness of specialties that non-volunteers matched to was 59.3 (IQR 23.7). The Wilcoxon Rank Sum test showed no significant difference between the two groups (*p* = 0.061) (Table 2). Similarly, the median competitiveness rating of specialties chosen by executive officers was 39.9 (IQR 27.8) and did not differ significantly from that of volunteers who were not executive officers (58.4, IQR 23.7; *p* = 0.164) or non-volunteers (59.3, IQR 23.7; *p* = 0.067) (Table 2).

Spearman’s correlation was performed to compare the number of shifts worked by ICC volunteers to the competitiveness rating of their eventual specialty choice. There was no significant correlation (*p* = 0.335), and the correlation coefficient was very weak and negative (r_s_ = -0.061).

## Discussion

In this retrospective study, students who volunteered with the ICC were 29% more likely to match into a primary care specialty than students who did not volunteer (RR 1.29, 95% CI 1.05–1.59). Executive officers at the ICC were more than twice as likely to match into family practice compared to volunteers who were not executive officers (RR 2.41, 95% CI 1.10, 5.26) or non-volunteers (RR 2.27, 95% CI 1.05–4.89). However, in a post-hoc analysis it was determined that this difference was most likely attributable to executive officers volunteering more frequently compared to non-executive officers. When stratified by number of shifts worked, the proportion of executive officers who matched into family practice was no different from non-executive officer volunteers. Furthermore, in multiple logistic regression, number of shifts worked was significantly correlated to matching into family practice (*p* = 0.042) while executive officer status was not (*p* = 0.247). In fact, each additional shift volunteered was associated with a 3.9% increase in the odds of matching into family practice when adjusting for executive officer status as a potential confounder. There was no correlation between volunteering and competitiveness of specialty choice.

These results expand upon two previous studies that have demonstrated a relationship between SRFC volunteering and student interest in primary care specialties. In a survey of 914 medical students at the University of California San Diego before and after volunteering with their SRFC, Smith et al. found that involvement with the SRFC improved student interest in primary care and student interest in working with the underserved. [14]. Limitations included the lack of a control group, and the subjective assessment of interest in primary care rather than an objective measure such as eventual specialty choice. Similarly, Campos-Outcalt et al. in 1985 demonstrated that volunteers at the University of California Davis SRFC were more likely to enter primary care residencies than non-volunteers. [15]. Their study was more objective, as it utilized student specialty choice by match lists as the outcome measure, but it is outdated and was limited in scope as only Hispanic medical and premedical students were analyzed.

On the other hand, several recent studies have found no significant relationship between volunteering with an SRFC and primary care specialty choice. However, these studies had notable limitations [16–19]. Brown et al. stated that their survey had a low response rate (39.8%) and may not have been representative of the volunteers at their clinic. [18] Similarly, Tran et al. suggested that that their conclusions were limited by a small sample size (136 students) and large standard deviations on survey responses. [16]. Vaikunth et al. conducted a more robust analysis in their study. They used objective measures such as alumni match statistics for specialty interest (rather than surveys) and incorporated student academic performance. However, their SRFC was explicitly focused on serving Hispanic populations, which may have selected for student volunteers with proficiency in Spanish. [17] Their resulting analysis was less representative of their whole medical school class. In addition, they included obstetrics/gynecology as a primary care specialty, unlike this investigation.

The present study used the quantitative outcome measure of alumni match statistics and did not rely on self-reported survey data. Our analysis included a large sample size of 506 students evenly distributed between the volunteer (50.2%) and non-volunteer (49.8%) cohorts. Additionally, our clinic does not target volunteers who speak a specific language and is highly representative of the entire medical student class at CMS. Finally, this analysis included several unique measures including leadership involvement, the number of shifts worked, and specialty competitiveness. The results of this investigation indicate that any SRFC involvement at our institution is associated with pursuit of primary care specialties, and degree of involvement is associated with pursuit of family practice. However, whether these associations represent a causative relationship requires further investigation.

There is some evidence in the literature supporting the notion that the associations seen in the present study are indeed causative. Having longitudinal primary care experiences has been reported to increase medical students’ likelihood of pursuing primary care specialties [20, 21]. Volunteering with the ICC, which provides primary care services to underserved patient populations, may constitute such a longitudinal primary care experience and thus influence students to pursue primary care. If this is the case, SRFCs may be a valuable tool to encourage interest in primary care specialties among medical students. However, it is worth noting that despite the increase in prevalence of SRFCs in the US over the past ~ 15 years, the shortage of primary care physicians continues to rise [1, 3–5]. Not all SRFCs focus on primary care services and provide their students with opportunities for longitudinal volunteering experience as our ICC does. As such, it remains possible that even if our ICC does have a causal impact on student interest in primary care, other SRFCs may not. Future research should explore not only whether the associations found in this study are indeed causal, but also whether they extend to all SRFCs or only SRFCs that have certain characteristics.

### Limitations

It is important to recognize the possibility that students with a pre-existing interest in primary care may have volunteered with the ICC at a higher rate compared to students with no interest in primary care, introducing a selection bias. On the other hand, most students who volunteer with the ICC are first- or second- year students and studies have shown that most medical students change their intended specialty over the course of medical school. [22, 23]. The AAMC reports that just 25.6% of medical school graduates went into the specialty they had indicated was their intended area of practice before entering medical school [22]. Compton et al. report that only 30% of medical students interested in primary care at first-year orientation remained interested through their fourth year of medical school. [23]. Thus, instability in medical student specialty preferences could have a mitigating effect on this type of selection bias if it is present.

Additionally, it is worth considering the quantification method used to approximate medical specialty competitiveness. Few studies have attempted to analyze competitiveness, and most have used fill rate as a proxy for competitiveness [8–11]. However, fill rate discounts student self-selection (i.e., medical students selecting which specialty they apply to according to the strength of their application). A better competitiveness measure should account for these variables, perhaps by including factors such as clerkship grades, board scores, and number of publications. Furthermore, associations between volunteering and performance in subsequent residencies was not evaluated in this investigation. Another limitation is that eventual specialty choice was defined using residency match data, no information regarding fellowships or further specialization was used. Therefore, some of the students categorized as matching into primary care specialties may go on to non-primary care specialties. Finally, the number of shifts worked by ICC volunteers was determined using shift schedules, which may not have included last minute changes such as volunteer cancellations or shift coverages.

## Conclusions

Medical students at Chicago Medical School who volunteer with the student-run free clinic are more likely to match into primary care residencies than non-volunteers. Students who volunteered more frequently were more likely to match into family practice specifically. Further investigation is warranted to determine whether these associations are causative and could thus be leveraged to encourage medical students to pursue primary care careers.

## Data Availability

The datasets used and analyzed during the current study are available from the corresponding author on reasonable request.

## Abbreviations

AAMC: Association of American Medical Colleges
CI: Confidence Interval
CMS: Chicago Medical School
ICC: Interprofessional Community Clinic
IQR: Interquartile Range
IRB: Institutional Review Board
NRMP: National Resident Matching Program
RR: Relative Risk
SRFC: Student-Run Free Clinic
US: United States
